# Improved reliability of serological tools for the diagnosis of West Nile fever in horses within Europe

**DOI:** 10.1371/journal.pntd.0005936

**Published:** 2017-09-15

**Authors:** Cécile Beck, Steeve Lowenski, Benoit Durand, Céline Bahuon, Stéphan Zientara, Sylvie Lecollinet

**Affiliations:** 1 University Paris Est, UMR 1161 Virology, ANSES, INRA, Ecole Nationale Vétérinaire d’Alfort, ANSES Animal Health Laboratory, EURL on Equine Diseases, Maisons-Alfort, France; 2 University Paris Est, ANSES Animal Health Laboratory, Epidemiology Unit, Maisons-Alfort, France; University of Texas Medical Branch, UNITED STATES

## Abstract

West Nile Fever is a zoonotic disease caused by a mosquito-borne flavivirus, WNV. By its clinical sensitivity to the disease, the horse is a useful sentinel of infection. Because of the virus’ low-level, short-term viraemia in horses, the primary tools used to diagnose WNV are serological tests. Inter-laboratory proficiency tests (ILPTs) were held in 2010 and 2013 to evaluate WNV serological diagnostic tools suited for the European network of National Reference Laboratories (NRLs) for equine diseases. These ILPTs were designed to evaluate the laboratories’ and methods’ performances in detecting WNV infection in horses through serology. The detection of WNV immunoglobulin G (IgG) antibodies by ELISA is widely used in Europe, with 17 NRLs in 2010 and 20 NRLs in 2013 using IgG WNV assays. Thanks to the development of new commercial IgM capture kits, WNV IgM capture ELISAs were rapidly implemented in NRLs between 2010 (4 NRLs) and 2013 (13 NRLs). The use of kits allowed the quick standardisation of WNV IgG and IgM detection assays in NRLs with more than 95% (20/21) and 100% (13/13) of satisfactory results respectively in 2013. Conversely, virus neutralisation tests (VNTs) were implemented in 33% (7/21) of NRLs in 2013 and their low sensitivity was evidenced in 29% (2/7) of NRLs during this ILPT. A comparison of serological diagnostic methods highlighted the higher sensitivity of IgG ELISAs compared to WNV VNTs. They also revealed that the low specificity of IgG ELISA kits meant that it could detect animals infected with other flaviviruses. In contrast VNT and IgM ELISA assays were highly specific and did not detect antibodies against related flaviviruses. These results argue in favour of the need for and development of new, specific serological diagnostic assays that could be easily transferred to partner laboratories.

## Author summary

The European network of National Reference Laboratories (NRLs) for equine diseases guarantees West Nile virus (WNV) surveillance and warning of the emergence of the disease. The WNV NRL network has gathered together most of the European countries facing WNV outbreaks. In this context, two inter-laboratory proficiency tests (ILPTs) were designed in 2010 and 2013 to evaluate the network’ and methods’ performances in detecting WNV infection through serology. A comparison of these two ILPTs emphasised a substantial improvement in the analytical performance of the WNV antibody detection tools over the years within the European NRLs network. Nevertheless the serological cross-reactions among related flaviviruses, such as the Japanese encephalitis, Usutu or tick-borne encephalitis viruses through IgG detection, associated with the Virus Neutralisation Tests’ (VNT) lower sensitivity, long duration and need for Biosafety Level 3 (BSL-3) facilities are major concerns related to indirect WNV diagnosis. All these remarks plead in favour of the development and implementation of new technologies to provide alternatives to classical methods for serological flavivirus diagnosis.

## Introduction

West Nile fever (WNF) is a zoonotic disease caused by West Nile virus (WNV). It can cause a severe neuro-invasive disease (WNND for WN neuro-invasive disease) in 1–10% of infected horses. WNF cases in horses have to be reported to international organisations (the World Organisation for Animal Health (OIE) and the European Commission for European countries). The virus is primarily transmitted by mosquitoes of the *Culex* genus and is amplified by wild bird reservoirs. WNV circulation has been regularly reported in the Mediterranean basin, as well as in Eastern and Central Europe since 1998. WNV outbreaks have increased substantially since 2010 in Europe [1]. The endemic circulation of WNV in several European countries (Romania, Italy and Spain) and regions (the Balkans) argue for reinforced surveillance of this disease [2].

WNV surveillance varies among European countries, ranging from clinical surveillance of horses or humans to active surveillance of birds or other infected species through regular serological screening and/or active WNV detection in trapped mosquitoes. This active surveillance is promoted in endemic areas where horses could have been previously infected or vaccinated against WNV [3]. Nevertheless, due to its clinical sensitivity to WNV infection, the horse is a sentinel whatever the surveillance system used. Horse cases of WNV can usually be diagnosed before human cases [4, 5]. In such a context, the improved detection of WNV infection in this species would be extremely helpful. In the case of WNND, WNV diagnosis is hampered by the short duration and low level of WNV viraemia. Consequently, serological diagnostic tests are preferred to confirm WNV infection in horses. The evidence of IgM antibodies in serum or cerebrospinal fluid (CSF) or the increase in Immunoglobulin G (IgG) titres in 2 serial samples obtained 2–3 weeks apart is sufficient to confirm WNV infection in horses. Many different serological tools are available to diagnose or screen for WNV antibodies [6]. The most commonly used are virus neutralisation tests (VNTs), including the plaque reduction neutralisation test (PRNT) or micro-virus neutralisation test (micro-VNT), immunofluorescence assays (IFAs), and enzyme linked immunosorbent assays (ELISAs). While rapid tests such as ELISAs and IFAs are preferred because of their sensitivity, reproducibility and affordability, WNV VNTs are still gold standard tests and offer high diagnostic specificity.

Noteworthy, seropositive tests should be interpreted with care due to frequent cross-reactions among flaviviruses, especially in rapid serological tests. Indeed, WNV belongs to the *Flavivirus* genus and many related flaviviruses are reported in Europe such as the mosquito-borne Usutu (USUV) and Bagaza viruses (BAGV), as well as the tick-borne encephalitis viruses (TBEVs), Louping ill virus (LIV) or Meaban virus [6–10]. Infection by such flaviviruses has been shown to induce antibodies that generate positive results in rapid serological diagnostic tests.

Recently, USUV—a flavivirus that originated in Africa—has been isolated in Austria, Belgium, the Czech Republic, Hungary, Italy, Germany, Greece, Poland, Switzerland and Spain. It has also emerged in France and the Netherlands in 2015 and 2016 respectively [8, 11–15]. BAGV—also termed Israel turkey meningoencephalitis virus (ITMV)—was first isolated in 1960 in Israeli turkeys with neurological symptoms, and has more recently emerged in Europe and more specifically in Spain in 2010, where it was found in partridges and pheasants [7].

TBEV and LIV are pathogens borne by *Ixodes* ticks. TBEV has been responsible for major encephalitis outbreaks in humans in northern Europe [16, 17]. LIV is a zoonotic pathogen causing encephalitis mainly in sheep and cattle, and is concentrated in Ireland and the United Kingdom [6, 18].

The risk of the emergence of new flaviviruses in Europe such as the Japanese encephalitis virus (JEV) or Zika viruses should not be neglected. JEV genomic RNA was detected in dead birds in Tuscany around 1997–2000 and in mosquitoes in Northern Italy in 2010 [19, 20].

Such a circulation of varied flaviviruses in Europe and the large cross-reactivity of rapid WNV serological tools should be considered when performing serological WNV assays on horses. All positive serological results using rapid assays should be confirmed by a more specific VNT with the viruses known to circulate in the area.

WNV surveillance in animals is supported by a network of cooperating laboratories in Europe. In 2008, the European Commission appointed the French agency for food, environmental and occupational health and safety (ANSES) as the European Reference Laboratory (EURL) for equine diseases with the remit of harmonising and improving the diagnosis of equine diseases in Europe. Two inter-laboratory proficiency tests (ILPTs) were performed in 2010 and 2013 by the network of European laboratories. This study was designed to acquire insights into the performance of serological methods and protocols applied in Europe to detect WNV (OIE manual, chapter 2.1.20) [21] and to evaluate the homogeneity of results among National Reference Laboratories (NRLs) in European and Mediterranean countries.

## Materials and methods

### Participating laboratories

**Fig 1:**
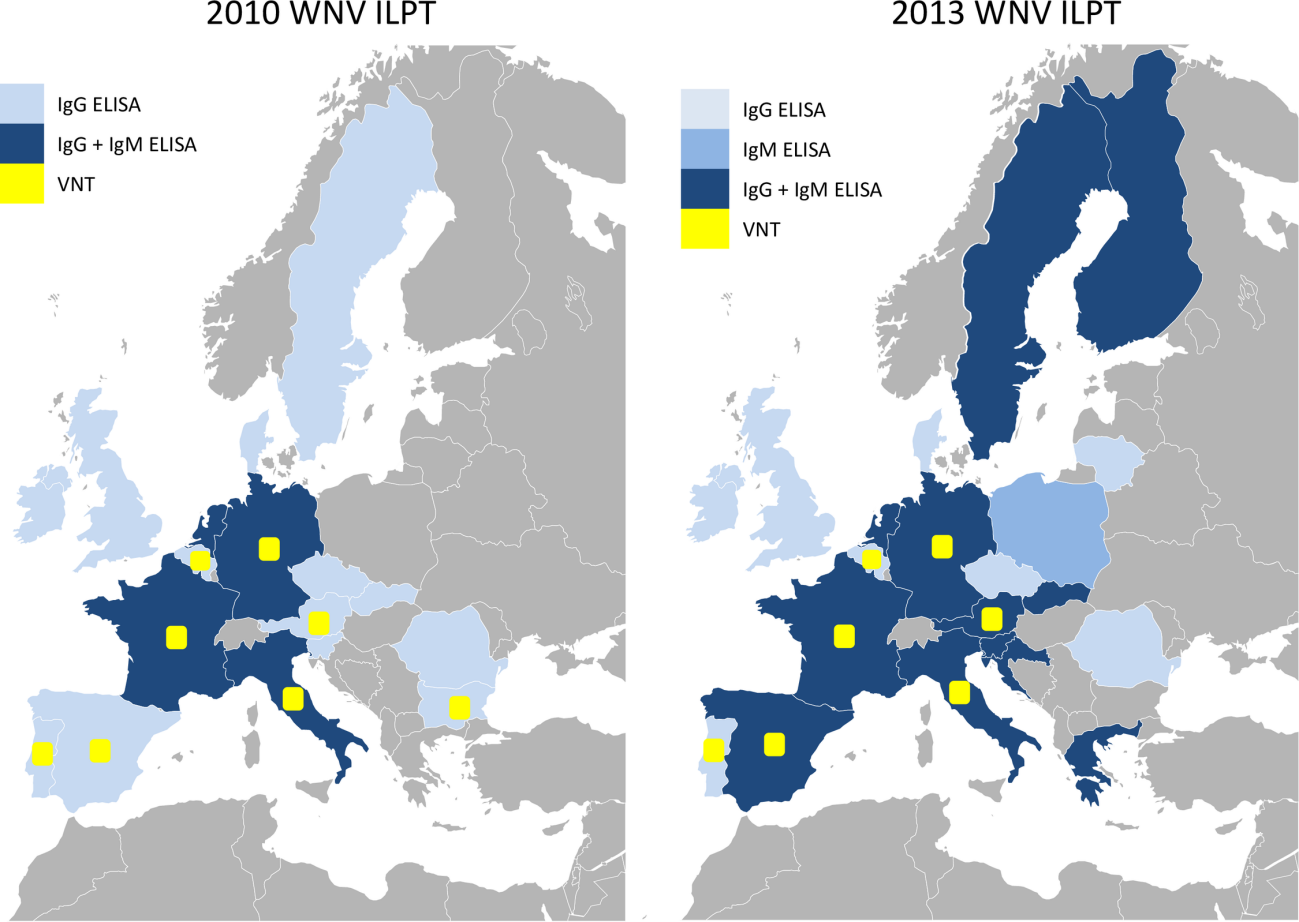
NRLs having participated in the 2010 and 2013 WNV ILPTs and serological diagnostic assays used. Diagnostic assays implemented in 2010 and 2013 by European NRLs are shown in pale blue (IgG ELISA alone), medium blue (IgM ELISA alone) or dark blue (IgG and IgM ELISAs) and in yellow (WNV VNT). (Base layer: lemondedesetudes.fr/freebies-cartes-powerpoint).

Seventeen European Union NRLs participated in the ILPTs on WNV serology in 2010 and 21 participated in 2013 (Fig 1 and Table 1). Participants also included reference laboratories from Morocco (in 2010 and 2013), Bosnia and Herzegovina (2013), other European participants (French, German, Irish, Italian or Spanish laboratories involved in WNV surveillance) as well as participants producing commercial WNV ELISA kits (1 in 2010 and 3 in 2013). In all, 21 and 35 participants were included in the 2010 and 2013 ILPTs respectively (S1 File). A number code was assigned to each participating laboratory to ensure a blind analysis of the ILPT results.

**Table 1:** Number of participants and serological methods used by participating laboratories during the 2010 and 2013 WNV ILPTs.

	Participants	Number	IgG ELISA kit	In-house IgG ELISA	IgM ELISA kit	In-house IgM ELISA	WNV VNT
**2010 ILPT**	NRLs	17	19*	1	3	1	8
	other European participants	2	1	1	1	1	0
	Kit manufacturers	1	0	1	0	0	0
	Mediterranean and Balkans NRLs	1	1	0	0	0	0
	**Total**	**21**	21*	**3**	**4**	**2**	**8**
**2013 ILPT**	NRLs	21	21*	1	13	0	7
	other European participants	9	4	2	7	1	2
	Kit manufacturers	3	2	0	2	0	0
	Mediterranean and Balkans NRLs	2	1	0	2	0	0
	**Total**	**35**	28*	**3**	**24**	**1**	**9**

*some participants used more than one kit. Ig: immunoglobulin; ILPT: inter-laboratory proficiency tests; NRLs: National Reference Laboratories; VNT: virus neutralisation test;WNV: West Nile virus

### Panel description

The panel of samples consisted of 16 and 15 sera in 2010 and 2013 respectively, with a minimum of 100 μl of horse serum per sample. Horse sera with or without antibodies directed against WNV or other flaviviruses were deliberately chosen. Samples were heat-inactivated at 56°C for 30 minutes, aliquoted and stored at -20°C until shipment to avoid degradation of the antibodies. The homogeneity of the serum preparation was confirmed on 5 panels by ELISA tests prior to shipment. Panels were randomly coded and shipped in dry ice to participants (Table 2).

**Table 2:** Description of the 2010 and 2013 ILPT panels.

		**2010-Serum**	**Serum dilution**	**Virus or antigen**	**Origin**	**Infection or dpv**
**2010 ILPT**	**Negative sera**	S1	NA	NA	Commercial horse serum	no
		S2	NA	NA	Commercial horse serum	no
		S3	NA	NA	Commercial horse serum	no
		S4	NA	NA	Commercial horse serum	no
	**Duplicate**	S5	1/20	Recombitek equine WNV	vaccination	35 dpv
		S6	1/20	Recombitek equine WNV	vaccination	35 dpv
	**Serial 2-fold dilutions**	S7	1/40	Recombitek equine WNV	vaccination	28 dpv
		S8	1/80	Recombitek equine WNV	vaccination	28 dpv
		S9	1/160	Recombitek equine WNV	vaccination	28 dpv
		S10	1/320	Recombitek equine WNV	vaccination	28 dpv
		S11	1/640	Recombitek equine WNV	vaccination	28 dpv
		S12	1/80	Recombitek equine WNV	vaccination	49 dpv
		S13	1/20	WNV-lineage2	Hungary (2008)	Natural Infection
	**Duplicate**	S14	1/40	WNV-lineage 1	Qatar (2005)	Natural Infection
		S15	1/40	WNV-lineage 1	Qatar (2005)	Natural Infection
		S16	1/10	WNV-lineage1	Southern France (2008)	Natural Infection
		**2013-Serum**	**Dilution**	**Virus or Antigen**	**Origin**	**Infection or dpv**
**2013 ILPT**	**Negative serum**	S1	NA	NA	Commercial horse serum	no
	**Kinetics of WNV lineage 1 antibody response**	S2	NA	WNV-lineage1, Israel 1998 strain	Experimental infection	8 dpi
		S3	NA	WNV-lineage1, Israel 1998 strain	Experimental infection	21 dpi
		S4	NA	WNV-lineage1, Israel 1998 strain	Experimental infection	35 dpi
	**Duplicate**	S5	1/6	WNV-lineage1, Israel 1998 strain	Experimental infection	35 dpi
		S6	1/6	WNV-lineage1, Israel 1998 strain	Experimental infection	35 dpi
	**Serial dilutions**	S7	1/6	WNV-lineage 2, Austria 2008 strain	Experimental infection	35 dpi
		S8	1/24	WNV-lineage 2, Austria 2008 strain	Experimental infection	35 dpi
		S9	1/48	WNV- lineage 2, Austria 2008 strain	Experimental infection	35 dpi
		S10	1/384	WNV- lineage 2, Austria 2008 strain	Experimental infection	35 dpi
		S11	1/384	WNV- lineage 2, Austria 2008 strain	Experimental infection	35 dpi
		S12	1/536	WNV-lineage 2, Austria 2008 strain	Experimental infection	35 dpi
	**Related flavivirus**	S13	NA	USUV, Italy 2012 strain	Experimental infection	35 dpi
	**Related flavivirus**	S14	1/3	JEV- Genotype 3, Nakayama strain	Experimental infection	35 dpi
	**Related flavivirus**	S15	1/2.6	TBEV, Hypr strain	Experimental infection	35 dpi

ILPT: inter-laboratory proficiency tests; WNV: West Nile virus; JEV: Japanese encephalitis virus; TBEV: Tick-borne encephalitis viruses; NA: not applicable; dpv = days post-vaccination; dpi = days post-infection

Participants were given 4 weeks to send back their results and were asked to store the samples either at -20°C or 4°C and to avoid repetitive thawing/freezing cycles. The stability of the panels at the end of the ILPT period was tested on one panel stored at 4°C throughout the ILPT period.

#### 2010 sera panel

Positive sera consisted of field sera and sera from vaccinated horses. Field sera were obtained during WNV outbreaks in Qatar in 2005 (lineage 1 WNV strain) and in Hungary in 2008 (lineage 2 WNV strain), as well as from serosurveys (Southern France, 2008) [22, 23]. Moreover, sera from horses vaccinated with recombinant Canarypox vector including prM and E WNV genes (Recombitek Equine West Nile Virus, Merial) and sampled 28, 35 or 49 days after the start of the primary and booster vaccination protocol (dpv) were included.

#### 2013 sera panel

The panel was composed of horse sera from experimental infections with different flaviviruses (WNV lineage 1 and WNV lineage 2, USUV, JEV and TBEV). The sera from experimental infections were obtained from 5 Welsh mares infected subcutaneously with 10^7^ pfu of either WNV lineage 1 (WNV1, IS-98-ST1 or Israel 1998 strain) or lineage 2 (WNV2, Austria 2008 strain), JEV (Nakayama strain), TBEV (Hypr strain) or USUV (Italy 2012 strain) and collected 8, 21 and 35 days post-infection (dpi) as described in [24].

The composition of the panels in 2010 and 2013 are described in Table 2. Positive and negative sera, duplicates of positive sera to analyse the reproducibility of the method plus serial dilutions of a positive serum to evaluate the dose-effect relationship were systematically included. In the 2013 panel, the kinetics of anti-WNV antibody responses with undiluted samples from a horse infected with WNV lineage 1 and sampled on days 8, 21 and 35 post-infection was also evaluated.

The analytical performances of participants were analysed according to the following criteria. Firstly, laboratories should have satisfactory sensitivity; the positive WNV IgG and IgM samples should be identified as positive. Secondly, the negative sera should be found to be negative. For the sera containing cross-reactive antibodies to heterologous flaviviruses (TBEV, USUV, JEV), different results were anticipated depending on ELISA performance (specificity), but negative results were expected by VNT. Thirdly, satisfactory reproducibility on 2 pairs of sera and coherent dose-effect curves on a serially diluted serum were expected.

### Serological methods

The participants were asked to test the samples with the diagnostic methods they routinely applied in their laboratories. They could use OIE- or EURL-recommended methods, commercial kits according to the manufacturer’s instructions or in-house methods.

The OIE gold standard serological assay is the PRNT with a threshold plaque reduction level of 90% (PRNT90) [21]. The micro-VNT is a modification of the PRNT and allows a larger number of samples to be screened using cell microplates [25].

Different ready-to-use diagnostic ELISA kits for veterinary purposes were commercially available. Two commercial competition kits—the ID screen WNV competition kit (ID Vet) and the Ingezim WNV Compaq kit (Ingenasa)—had been developed before 2010 and could be used to detect anti-E antibodies in multiple species. Both ELISA kits use plates pre-coated with WNV recombinant antigens and measure the competition between antibodies present in the animal serum tested and a monoclonal anti-E antibody conjugated to horseradish peroxidase (HRP). These competitive ELISA kits detect virtually every Ig isotype, but are mainly used to detect IgG and are classified herein among the IgG detection tools.

IgMs are specifically detected by IgM Antibody Capture ELISAs (MAC-ELISA). Equine anti-WNV IgMs present in horse sera bind to anti-horse IgM antiserum coated on ELISA plates. This binding is revealed by the addition of a positive antigen (recombinant WNV antigen), a monoclonal antibody directed against WNV antigen and conjugated to HRP and a chromogenic substrate. While the IDEXX IgM WNV Ab kit was available prior to 2010, the Ingezim WNV IgM and ID screen WNV IgM capture kits were marketed after 2010, so were only used during the 2013 ILPT.

### Statistical analyses

A statistical analysis on 2010 and 2013 ILPT results was carried out on the kits used for both ILPTs, to analyse the effect of the country and/or the effect of the batch on the result. A linear mixed model was used. The results obtained, *i*.*e*. the %S/N values with the ID screen WNV competition kit or ISR values with the IDEXX IgM WNV Ab kit, was the dependent variable. The country and the batch number were treated as fixed effects, the serum number being treated as a random effect. Log-likelihood tests were used to analyse the effect of the country and of the batch. This analysis was performed separately on results obtained for the detection of the anti-E antibodies (ID screen WNV competition kit was the only kit used in both ILPTs) and on results obtained for IgM detection (IDEXX IgM WNV Ab kit being the only test used in both ILPTs). Statistical analyses were conducted using R (R-Core Team, 2015) [26].

## Results

### Overall NRL network results

The number of European NRLs participating in these WNV ILPTs increased from 17 in 2010 to 21 in 2013. With such a condensed network of laboratories dedicated to WNV detection in animals, European countries should rapidly evidence WNV outbreaks. All the participants used commercial competition kits to detect anti-WNV antibodies in both ILPTs. Only 6% of NRLs (1/17) in 2010 and 5% (1/21) in 2013 used an in-house IgG indirect ELISA (with inactivated virions) in addition to the commercial competition kit.

Following the commercialisation of horse WNV MAC-ELISAs, the number of NRLs detecting anti-WNV IgM antibodies clearly increased between 2010 (18% (3/17) NRLs) and 2013 (62% (13/21)). An in-house IgM ELISA was used by only one NRL in 2010. In contrast, the WNV VNT—which requires the handling of infectious WNV in BSL-3 facilities—was not implemented in new NRLs between 2010 (8/17) and 2013 (7/21) (Fig 1 and S1 File).

ELISAs are the preferred screening tools because of their rapidity, high throughput and sensitivity. Moreover, competitive ELISA kits in veterinary laboratories offer the possibility of testing sera from various animal species. During the 2010 and 2013 WNV ILPTs, the ID screen WNV competition kit was used most often (90% (19/21) of assays in 2013), while the Ingezim WNV Compaq kit was used by only two NRLs (2/21). These two kits detect antibodies against the WNV Envelope (E) glycoprotein that are induced after natural infection or vaccination (the WNV E structural antigen is expressed by all WNV vaccines marketed for horses) [24]. Nevertheless, depending on the WNV conjugate used in the kit (anti E-domain III in the Ingezim WNV Compaq kit or anti-E antibodies directed against less specific domains in the ID screen WNV competition kit), cross-reactions following infections with other flaviviruses were observed to a greater or lesser extent (see “results according to the method”).

The sensitivity of IgG detection methods was evaluated with samples containing high levels (S5, S6 and S13 in 2010, as well as S3-S7 in 2013) or low levels of WNV-IgG (S7 and S16 in 2010 and S8-S9 in 2013) (Table 2).

**Fig 2:**
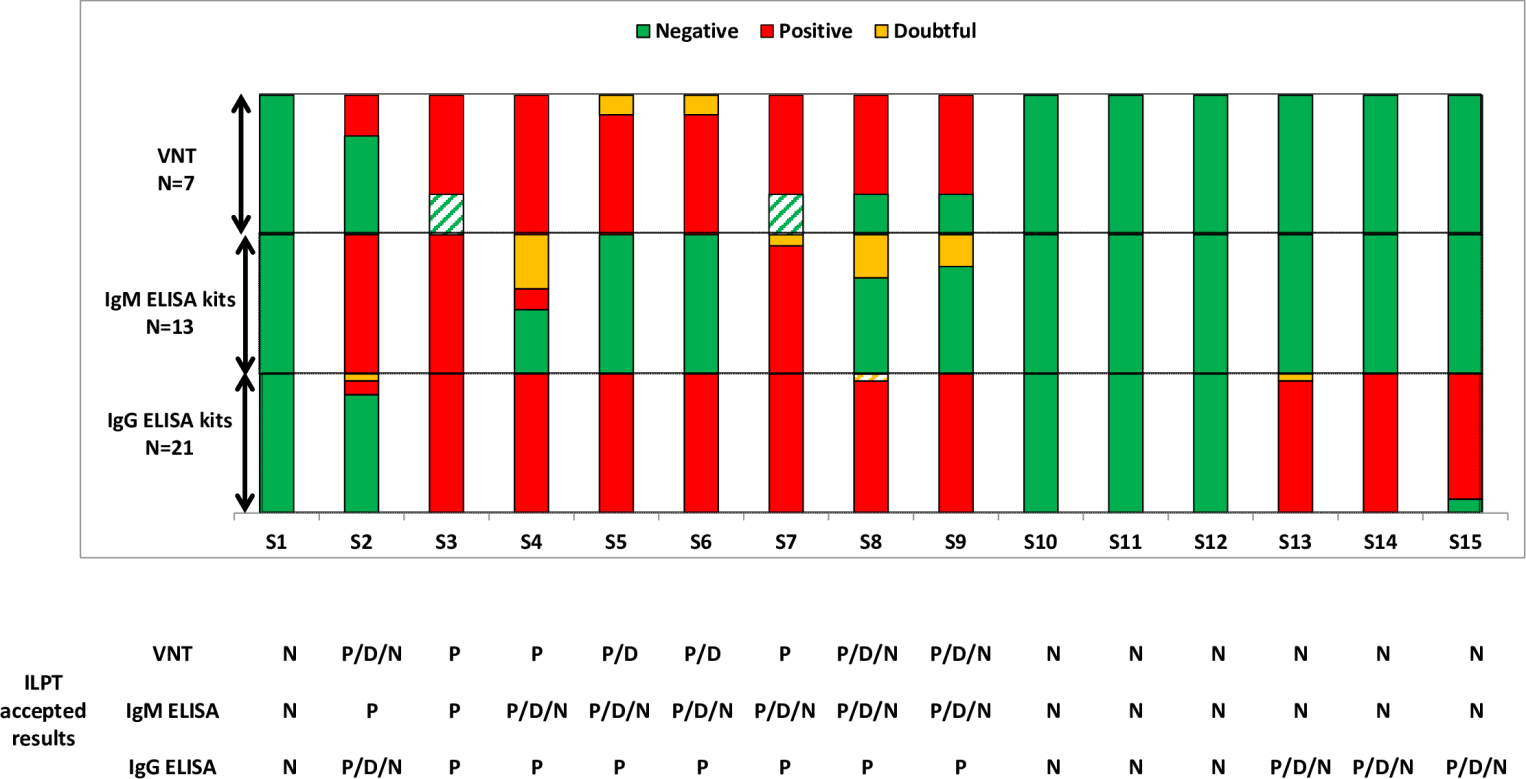
Percentage of NRLs having generated positive (red), doubtful (orange) and negative results (green) during the 2013 WNV ILPT. Doubtful are equivocal results; Striped results correspond to unsatisfactory results different from the accepted results. The below table summarises the accepted ILPT results taking into account the performance of the tests used by the participants with P (positive), D (doubtful) and N (negative).

Two participants in 2010 and only one participant in 2013 encountered sensitivity issues with commercial kits (Fig 2, S2 and S3 Files). The in-house indirect IgG ELISA (participant E, S2 File) clearly lacked sensitivity in 2010, producing 4 false negatives, but an improvement in its sensitivity was observed in 2013 with only one positive sample generating a doubtful result (participant E, S3 File).

The specificity of IgG methods was first evaluated on negative horse sera (S1-S4, S10 and S11 in 2010). In 2010, no cross-reactive antibodies to heterologous flaviviruses were present in the panel. The specificity was 100% with 6 negative horse sera found negative by all the participants.

In 2013, samples with antibodies against TBEV, USUV and JEV were added to the panel. Due to the fact that the monoclonal antibody used in some commercial kits recognises flaviviruses other than WNV, the test specificity was first evaluated on horse sera negative for anti-flavivirus antibodies (S1, S10-S12). The specificity was 100% with 4 negative horse sera found negative by all the participants using either commercial or in-house ELISAs (Fig 2).

Test reproducibility and coherent dose-effect curves were successfully achieved by every NRL in 2010 and 2013, with the exception of a single participant in 2010 (participant H) who obtained differing results on 2010-S5 and S6 duplicate samples.

Taken together, the NRL network obtained very good qualitative results, with 14/17 (82%) NRLs in 2010, and 19/20 (95%) in 2013 achieving satisfactory sensitivity, specificity and reproducibility.

**Fig 3:**
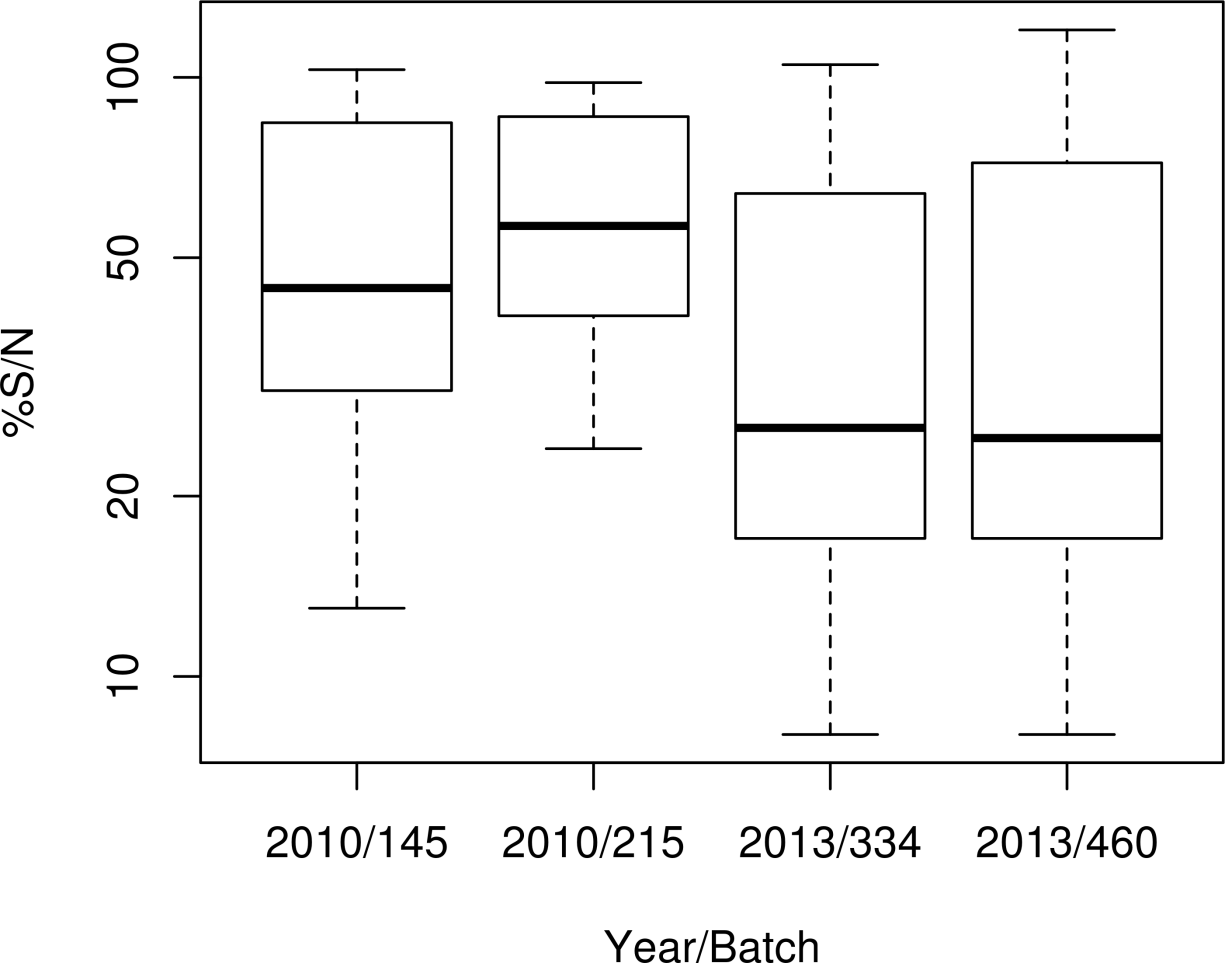
Box plot representing the distribution of %S/N obtained with the ID screen WNV competition kit by NRLs during the 2010 and 2013 ILPTs according to the year and batch. Assays were performed and %S/N was calculated according to the manufacturer’s instructions (the threshold value for considering a serum as positive by the competitive ELISA was %S/N < 40%).

**Fig 4:**
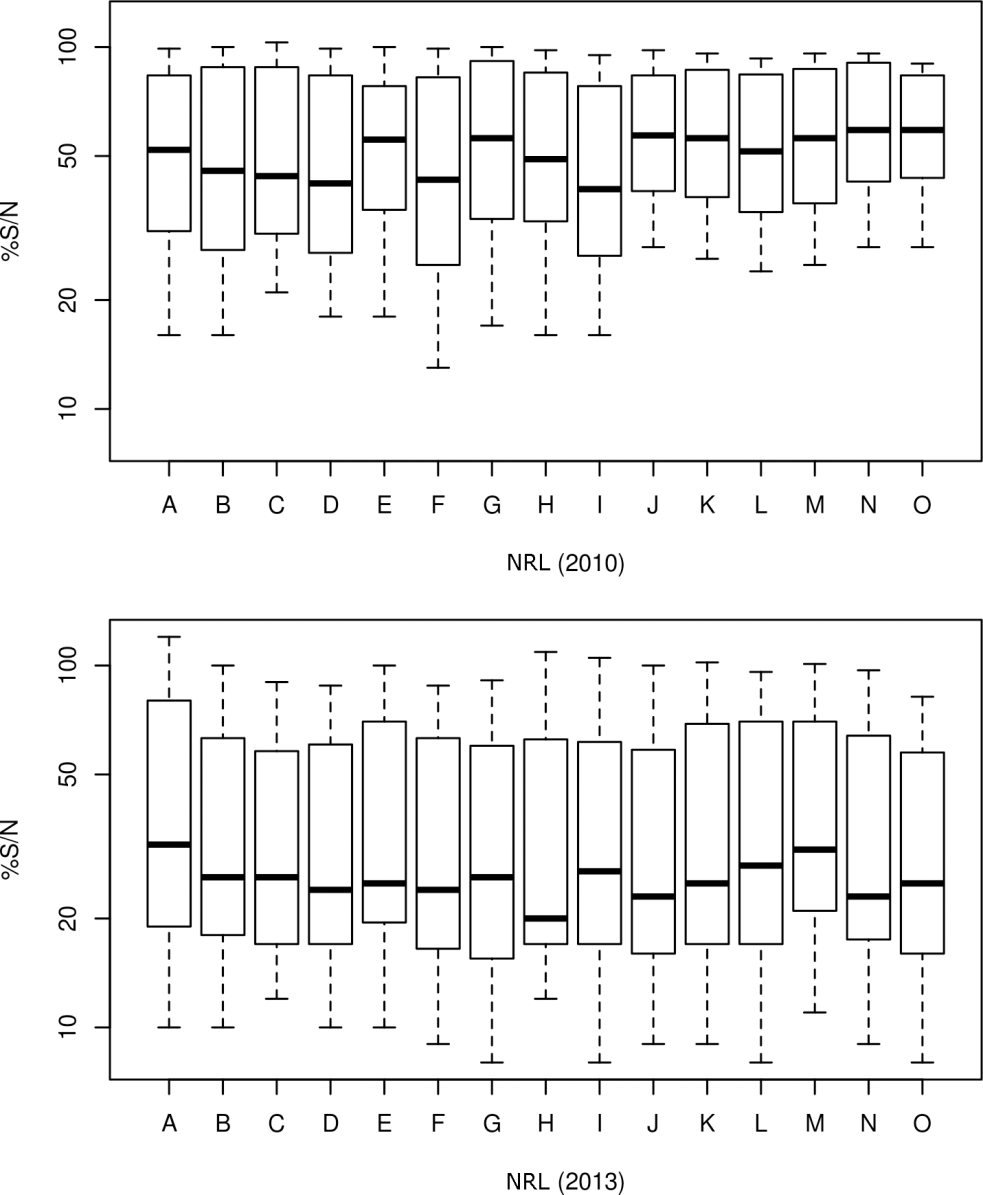
Box plot representing %S/N obtained with the ID screen WNV competition kit by each NRL during the 2010 and 2013 IPLTs. A single letter, A-0, refers to a unique NRL in 2010 and 2013.

A statistical analysis on 2010 and 2013 results was carried out on the only kit used for these 2 ILPTs (*i*.*e*. the ID screen WNV competition kit), to analyse the effect of the country and/or effect of the batch on the result. This analysis revealed that the %S/N values (on a log scale) varied significantly according to the NRL (p<0.0001) and to the kit batch (p<0.0001) (Figs 3 and 4). Both effects were observed when considering 2010 data (country: p = 0.0001, batch: p<0.0001), whereas the batch effect was not evidenced when considering data from 2013 (country: p<0.0001, batch: p = 1). Indeed, in 2010, batch 215 was clearly less sensitive than batch 145, the S14-S15-duplicate sample, for example, being found mostly positive with batch 145 but mostly negative with batch 215. In contrast, in 2013, the distributions of %S/N results obtained using batches 334 and 460 appeared similar (Fig 3).

MAC-ELISAs were used to detect immune response associated with acute and recent viral infection. The secretion of anti-WNV IgM can generally be detected as early as 2 to 8 days after the onset of initial clinical signs (fever).

Three NRLs in 2010 and 5 in 2013 used the IDEXX IgM WNV Ab kit, while 8 other NRLs used the ID screen WNV IgM capture in 2013; only 1 participant in 2010 used an in-house MAC-ELISA.

Sensitivity was assessed against 2 IgM-positive horse sera in 2010 (S14-S15-duplicate samples generated during a recent WNV infection) and 2 IgM-positive sera in 2013 (S2 and S3) produced by experimental infection.

Specificity was evaluated on the same samples as for the IgG ELISA tests (6 negatives in 2010 and 4 negatives in 2013, plus 3 sera produced after experimental infection with JEV, USUV and TBEV in 2013).

The NRL network achieved 100% specificity and 100% sensitivity in 2010 and 2013 with commercial and in-house MAC ELISAs (S2 and S3 Files).

A statistical analysis on 2010 and 2013 results from the IDEXX IgM WNV Ab kit (the only test used during both the 2010 and 2013 ILPTs) did not show a country or batch effect (p>0.05).

VNT is the gold standard serological tool recommended by the OIE Manual of Diagnostic Tests and Vaccines for Terrestrial Animals. This method offers higher specificity but appears less sensitive than ELISAs and is usually used as a confirmation and a titration method for WNV-specific neutralising antibodies (from serum or cerebrospinal fluid, CSF) [27].

In all, 8 NRLs in 2010 and 7 NRLs in 2013 used either the PRNT90 or micro-VNT on Vero cells with various WNV isolates (depending on NRLs, strains generally belonging to lineage 1—Eg101, Is98 or NY99—were used) (S3 File). Apart from the EURL, no other NRL used USUV, TBEV or JEV isolates to evidence antibodies against related flaviviruses.

All the participants achieved specific results with no false positives for true negative sera or for sera from horses infected with related flaviviruses. A lack of sensitivity was underlined in 3/8 NRLs in 2010 and in 2/7 NRLS in 2013, with 2 participants missing at least two positive samples in 2010 (S14-S15 duplicates) and 2013 (S3 and S7).

### Results according to the method

These WNV ILPTs were also used to evaluate the characteristics and performance of the different serological assays used by participating laboratories in terms of early detection of anti-WNV antibodies, sensitivity and specificity (observation of cross-reactive reactions with sera from horses infected with related flaviviruses).

Because fewer commercial kits for the detection of anti-WNV IgG and IgM were used in 2010 than in 2013, the results generated by competitive ELISAs, MAC ELISAs and VNTs were compared only for the 2013 ILPT. The assays used by 35 participating laboratories (21 NRLs and 9 other EU laboratories, 3 kit manufacturers and 2 Mediterranean and Balkans reference laboratories) are shown in Table 1 and analysed hereafter. The comparative results obtained by the analysis of ILPT results are purely indicative and do not entirely reflect the performances of the methods.

#### Early detection of anti-WNV antibodies

The early detection efficacy of MAC ELISAs (detection of IgM only), VNT and competitive ELISAs was evaluated.

Twenty-four results were obtained for the detection of WNV IgM antibodies (16 with the ID screen WNV IgM capture kit, 7 with the IDEXX IgM Ab capture kit and one with the Ingezim WNV IgM kit). Animals recently infected by WNV were all correctly detected as early as 8 dpi for the WNV lineage 1-infected pony (S2), whatever the commercial method used (S3 File).

By comparison, WNV VNT allowed the early identification of WNV-infected horses (S2 collected at 8 dpi) only by 3/9 NRLs.

Twenty-eight results were considered for the commercially available competitive ELISA kits (25 participants using the ID screen WNV competition kit and 3 the Ingezim WNV Compaq kit).

The kinetics of antibody response after WNV infection were studied on undiluted samples from a WNV lineage 1-infected horse, sampled on 8 (S2), 21 (S3) and 35 (S4) dpi. Antibody detection was effective as early as 8 dpi with the Ingezim WNV compaq kit (3/3 assays and 2 different batches). This kit appeared to detect recently-infected animals more efficiently than the ID screen WNV competition kit which evaluated sample S2 sampled on 8 dpi as negative in 23/25 assays and doubtful in 2/25 assays. However, on 21 dpi, WNV antibodies were detected whatever the kit used (S3 File).

#### Analytical sensitivity of the methods

The competitive ELISA kits were found to have a high analytical sensitivity. Samples containing low levels of anti-WNV IgG like S9 diluted to 1/48 were still found to be positive by the 2 commercial ID screen and Ingezim WNV competition kits. Analytical sensitivity was equivalent with both commercial kits.

As regards MAC ELISA, the Ingezim WNV IgM kit seemed to offer a higher analytical sensitivity. Indeed, the duplicate S5 and S6 samples corresponding to a serum sampled on 35 dpi from the WNV lineage 1-infected pony and diluted to 1/6 were found positive with the Ingezim WNV IgM kit (one assay) and negative with IDEXX IgM WNV Ab (7 assays) and ID screen WNV IgM capture kits (16 assays). Moreover, S8 and S9 sera sampled on 35 dpi from the WNV lineage 2-infected pony and diluted to 1/24 and 1/48 respectively was twice found positive with the Ingezim WNV IgM kit (1/1 assay), doubtful for 5/7 assays with IDEXX IgM WNV Ab and negative with ID screen WNV IgM capture kits (16/16 assays).

VNT results varied depending on the participant performing the assay, with 3/9 participating laboratories not detecting low positive samples S8 (1/24) and S9 (1/48).

#### Cross-reactivity of WNV serological assays

During the 2013 ILPT, the Ingezim WNV Compaq kit appeared to be more specific than the ID screen WNV competition kit and failed to detect TBEV-positive sera. Serum from a TBEV-infected pony (S15) was not detected with the Ingezim WNV Compaq kit (3/3 assays) and generated positive cross-reactions (24/25 assays) with the ID screen WNV competition kit. These results should be confirmed by further studies with the Ingezim WNV Compaq kit on TBEV-positive sera.

Nevertheless, when considering horses infected with flaviviruses belonging to the same serocomplex as WNV *i*.*e*. USUV (S13) and JEV (S14), horses were found positive or doubtful by 100% (28/28) of the participants whatever the ELISA kit used (S3 File). False positive reactions with related flaviviruses are clearly mentioned on the leaflet of the ID screen WNV competition kit.

Interestingly, an in-house anti-NS1 competition assay carried out in the 2013 ILPT (1 assay) appeared to be more specific than anti-E competitive kits, detecting WNV- and JEV-positive sera only (participant AD, S3 File). The best specificity was achieved with two in-house indirect or competitive ELISAs (participant numbers E and AB respectively; see S3 File) with no cross-reactivity revealed for related flaviviruses (2 assays).

WNV PRNT or micro-VNT more specifically detected WNV antibodies. No cross-neutralisation was observed in particular on sera from horses infected with viruses belonging to the JEV serocomplex such as USUV or JEV (S3 File).

MAC-ELISAs were more specific than IgG indirect or competitive ELISAs, and generated negative results for all the sera sampled from horses recently infected with related flaviviruses (S13, S14 and S15; S3 File).

#### Distinction between vaccinated and infected horses

This analysis could be carried out only on the 2010 ILPT panel consisting of field sera and sera from horses vaccinated with the Recombitek Equine West Nile Vaccine produced by Merial. The duplicate sera S5 and S6 produced by the vaccinated horse and diluted to 1/20 were systematically found positive with the two competitive ELISA kits (S2 File). We obtained negative results for these same samples (S5 and S6) with an in-house ELISA targeting specific NS1 epitopes suggesting that the Recombitek Equine West Nile Vaccine elicited anti-E but not anti-NS1 antibody responses [28]. On the other hand, naturally-infected horses were found positive whatever the target antibody (anti-E or NS1 ELISAs).

## Discussion

The NRL network guarantees WNV surveillance and warning of the emergence or re-emergence of the disease. The number of laboratories has increased between the two ILPTs and indeed has been continuously increasing since 2008. Hungary, Latvia and Bulgaria were integrated as new NRL participants in addition to the 2013 network in an ILPT held in late 2016. A comparison of the two ILPTs organised in 2010 and 2013 demonstrated an improvement in the performances of WNV analytical assays and processes in 2013. Moreover, at least 13 NRLs are able to diagnose acute WNV infection and many of them (Greece, Austria, Italy, Croatia and France) were involved in the detection of recent WNV outbreaks [29]. In 2013, the WNV NRL network had gathered together most of the EU countries facing WNV outbreaks and EU countries with the highest equine population (Romania, Benelux countries, the United Kingdom, Germany and France) [30, 31]. Other European and international initiatives (H2020 MediLabSecure project, IAEA and FAO training initiatives) have fostered enhanced, more widespread WNV surveillance in other European countries and regions (such as the Balkans and the Mediterranean basin area).

Competitive ELISA kits are widely used to detect IgG antibodies against WNV. They are well established in Europe, with 17 NRLs and 20 NRLs using competitive WNV kits in 2010 and 2013 respectively. Thanks to the development of new commercial MAC ELISAs, ELISAs ensuring the early detection of infected horses have been rapidly implemented in reference laboratories between 2010 (3 NRLs using MAC ELISA kits) and 2013 (13 NRLs using MAC ELISA kits). The use of kits has allowed rapid standardisation of the 2 methods in NRLs, with more than 95% (20/21) and 100% (13/13) of relevant results in competitive and MAC ELISAs respectively in 2013.

An improvement in the sensitivity of competitive ELISA kits used by the NRL network was evidenced between the 2 ILPTs, with fewer false negative results generated during the 2013 ILPT. The specificity of competitive and MAC ELISAs on sera negative to flavivirus antibodies was 100% for the 2 ILPTs.

In European laboratories seeking to detect human WNV cases, only a few perform the WNV VNT [32]. An identical profile was observed in veterinary laboratories where a low but stable number of NRLs performed VNTs. The need for a BSL-3 facility and technique constraints can account for such an observation. Moreover, NRL results underlined the difficulties in standardising WNV VNTs, with a lack of sensitivity in 3/8 NRLs in 2010 and again in 2/7 NRLs in 2013.

Indirect or competitive WNV ELISAs are commonly used for screening purposes because of their reputed higher sensitivity [6]. The analytical sensitivity of these methods was evaluated in our ILPT with different WNV-positive sera originating from lineage 1 or 2 WNV-infected ponies and containing different levels of antibodies. Their sensitivity was shown in most cases to be higher than that of VNT, depending on the method [24] implemented by the NRL and the VNT’s own analytical sensitivity. Nevertheless, the false positives generated through cross-reactions with antibodies directed against heterologous flaviviruses confound the interpretation of ELISA tests. Specificity issues and the detection of infections caused by close flaviviruses have been systematically described in international quality control assessments for the human serological detection of WNV or Dengue virus (DENV) infections [32–34]. For example, high WNV IgG false positives were reported for samples containing anti-DENV antibodies [32]. Specifically, the ILPT organised in this study in 2013 showed that an infection by closely related flaviviruses belonging to the JEV serocomplex (*i*.*e*. USUV and JEV) systematically generated false positive reactions with the two commercially available competitive ELISAs, while kit specificity was more variable for flaviviruses belonging to another serocomplex (*i*.*e*. TBEV). Interestingly, the last ILPT organised in 2016 showed an improvement in the diagnostic specificity of the Ingezim WNV Compaq kit with USUV and JEV positive sera generating negative results with this kit (6/6 assays) [35]. Moreover, some ELISA protocols using alternative antigens (NS1) or revelation systems proved to offer higher specificity than commercial ELISA kits.

Indirect or competitive ELISAs are useful for the rapid screening of animal samples, keeping in mind that positive results are not indicative of WNV infection. In the event of positive results, the diagnosis of an acute WNV infection in horses should be confirmed by IgM detection in serum or cerebrospinal fluid.

MAC ELISAs are used to detect acute infections in humans and horses. Nevertheless, their usefulness in determining acute infections in humans is questionable because IgM response is sometimes detectable up to 1 year after initial virus exposure in plasma/serum [36]. In horses, IgM antibodies are short-lived [37].

In equids, WNV IgM antibodies are secreted as early as 8 dpi [27] and can be detected up to 70–90 dpi with currently developed IgM ELISAs [37]. With 42% of participants generating negative results on a WNV lineage 1 serum sampled at 35 dpi (S4), the window during which anti-WNV IgM antibodies were detected in the serum by MAC-ELISA seemed more limited than described in the literature. However, precaution should be taken when interpreting these data because the samples originated from experimental infections associated with asymptomatic or very mild infection. Moreover, the variable sensitivity of MAC ELISA commercial kits can also account for such a result, since the Ingezim WNV IgM kit generated a positive result on 35 dpi for the WNV lineage 1 sample (S4) as well as for the lineage 2 sample diluted to 1/48 (S9). These data should be confirmed with additional samples and assays calibrated for the evaluation of kit performances.

Finally MAC ELISAs appeared to be more specific than indirect IgG or competitive ELISAs [38]. Samples from USUV-, TBEV- and JEV-infected ponies were found by MAC ELISA to be negative on 35 dpi, while clearly positive by competitive ELISA kits (Fig 2). IgM antibodies against related flaviviruses also remained undetected in non-diluted sera sampled on 8 dpi and 21 dpi (S1 Fig). The short duration of anti-flavivirus IgM response in horses, associated with the high specificity of MAC ELISAs indicate that MAC ELISAs can be used to confirm recent WNV infection in horses [38]. Nevertheless, the production of IgM antibodies following primary vaccine administrations is questionable. Indeed, some papers in the literature consider IgM reactivity towards an inactivated vaccine as minimal [39] while others support low but detectable IgM antibody production [40]. Vaccination history should always be considered prior to the interpretation of MAC ELISAs.

**Fig 5:**
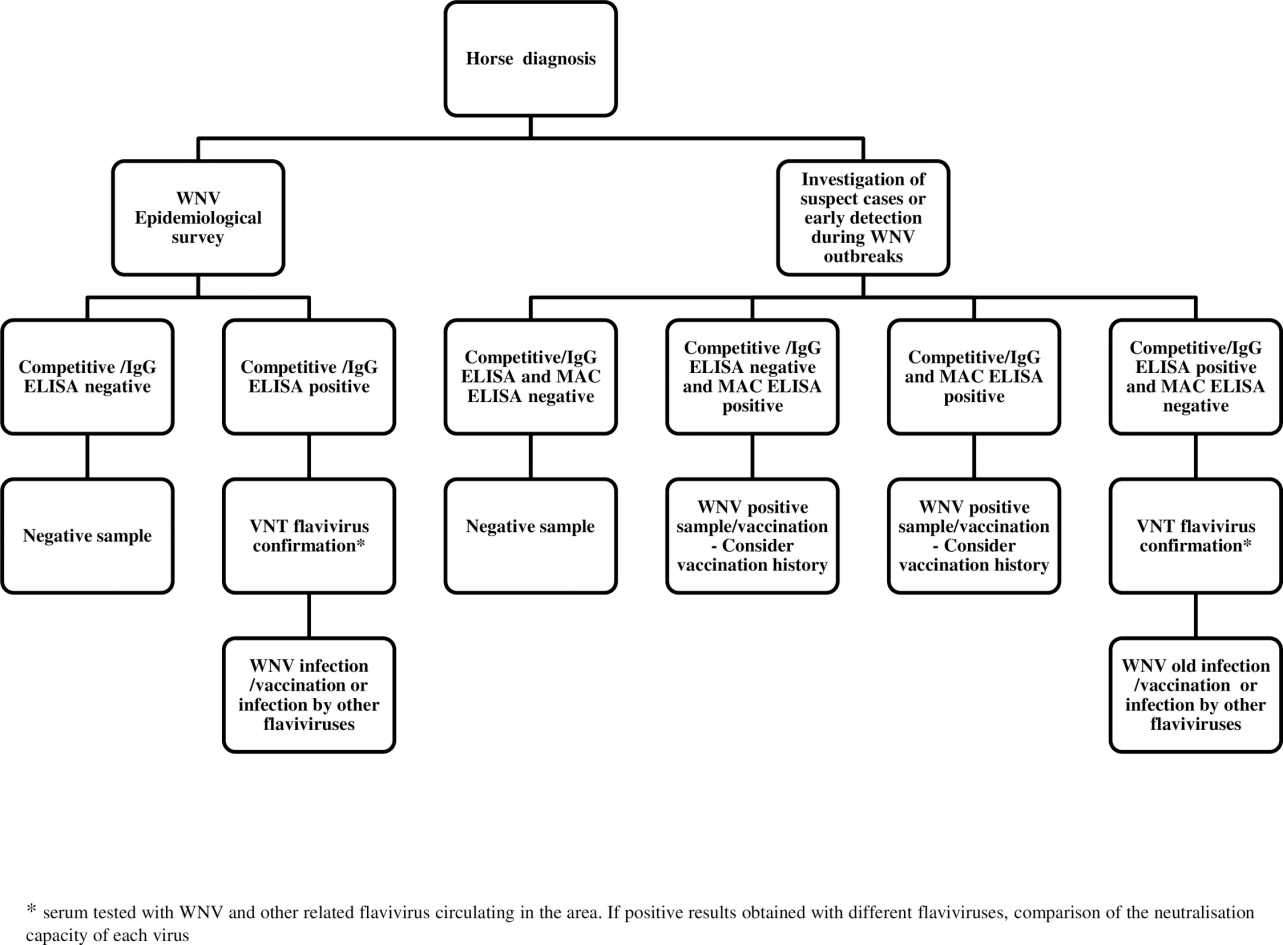
Decisional algorithm for flavivirus serological diagnosis in case of negative results by molecular diagnosis.

VNT is the gold standard serological tool for confirming WNV diagnosis [21]. In the WNV 2013 ILPT, WNV VNTs demonstrated variable sensitivity depending on the laboratory, but a high level of specificity with no false positives obtained with sera from horses infected with related flaviviruses. Nevertheless, cross-neutralisation by antibodies directed against viruses within the same serocomplex can still be observed in the field [41] despite no such evidence during the ILPT. Taking into account these conclusions, a decisional algorithm for serological flavivirus diagnosis is proposed in Fig 5.

All these remarks plead in favour of the development and implementation of new technologies to provide alternatives to classical methods for serological flavivirus diagnosis [42]. Many efforts have been made to develop ELISAs with enhanced specificity by using monoclonal antibodies targeting specific epitopes on NS1 or the E glycoprotein of flaviviruses [43, 44]. The ELISA targeting epitopes on NS1 appeared more specific for related flaviviruses in the 2013 ILPT, as also evidenced in the studies by Kitai et al. for differentiating JEV from WNV infection [45]. Moreover, this ELISA was able to distinguish infected horses from horses vaccinated with recombinant prM-E WNV vaccines (not containing the NS1 antigen) [46, 47]. Such a DIVA test could be useful in areas where horses have a high vaccination coverage.

The WNV E glycoprotein is folded into three structural domains: DI, DII and DIII. While DII is highly conserved among flaviviruses, DI and DIII contain virus-specific epitopes [48, 49]. Recombinant envelope proteins bearing mutations in the conserved DII part [50] or domain III of the E glycoprotein [51] have been used as specific antigens in ELISAs [44, 52, 53]. The development of multiplex serological tools is also another promising approach. Microsphere-based immunoassays using domain III antigens [24] or protein microarrays with recombinant NS1 proteins of flaviviruses [40] have been shown to offer advantageous alternatives to VNTs because they can test different flaviviruses in parallel.

## Supporting information

S1 File Participating laboratories in the 2010 and 2013 WNV ILPT. (PDF)

S2 File Results of the 2010 WNV ILPT per participant (IgG, IgM and VNT). (PDF)

S3 File Results of the 2013 WNV ILPT per participant (IgG, IgM and VNT). (PDF)

S1 Fig Reference equine sera sampled from ponies infected by different flaviviruses (WNV lineage 1, WNV lineage 2, USUV, JEV and TBEV) collected on different days (D) after infection (D8, D21 and D35) and tested by ID screen WNV IgM capture kit. %S/P was calculated according to the manufacturer’s instructions. The threshold value for considering a serum as positive was % S/P greater than or equal to 45%.
(PDF)
