# Supplementary material for: Improved reliability of serological tools for the diagnosis of West Nile fever in horses within Europe
Source: PLoS Negl Trop Dis. 2017 Sep 15;11(9):e0005936. doi: 10.1371/journal.pntd.0005936 (PMC5617233; doi:10.1371/journal.pntd.0005936)
Supplement: S1 File — (PDF) [file pntd.0005936.s001.pdf]

S1 File: Participating laboratories in the 2010 and 2013 WNV ILPT

				2010			2013		
**Institute (NRLs)**	**Country**	**2010**	**2013**	**IgG**	**IgM**	**VNT**	**IgG**	**IgM**	**VNT**
AGES IVET-Mödling Institute for Veterinary Disease Control Mödling	Austria	x	x	x		x	x	x	x
CODA-CERVA	Belgium	x	x	x		x	x		x
National Diagnostic and Research Veterinary Medical Institute	Bulgaria	x		x		x			
Faculty of Veterinary Medicine	Croatia		x				x	x	
Department of Virology	Czech Republic	x	x	x			x		
National Veterinary Institute, Technical University of Denmark (DTU-Vet)	Denmark	x	x	x			x		
Finnish Food Safety Authority Evira	Finland		x				x	x	
ANSES	France	x	x	x	x	x	x	x	x
Friedrich-Loeffler Institut, Federal Research Institute for Animal Health	Germany	x	x	x	x	x	x	x	x
Ministry of Rural development and Food, Athens Center of veterinary Institutes	Greece		x				x	x	
Central Veterinary Research Laboratory	Ireland	x	x	x			x		
Istituto Zooprofilattico Sperimentale dell'Abruzzo e Molise ,"G.Caporale"	Italy	x	x	x	x	x	x	x	x
National Food and Veterinary Risk Assessment Institute	Lithuania		x				x		
National Veterinary Research Institute (NVRI)	Poland		x					x	
Instituto nacional de investigação agrária e veterinária (INIAV)	Portugal	x	x	x		x	x		x
Institute for Diagnosis and Animal Health	Romania	x	x	x			x		
State Veterinary Institute Zvolen	Slovakia	x	x	x			x	x	
NRL for Equine diseases	Slovenia	x	x	x			x	x	
Laboratorio Central De Veterinaria	Spain	x	x	x		x	x	x	x
SVA National Veterinary Institute	Sweden	x	x	x			x	x	
Wageningen Bioveterinary Research	The Netherlands	x	x	x	x		x	x	
Animal and Plant Health Agency	United Kingdom	x	x	x			x		
**Other European participants**									
Irish Equine Center	Ireland	x	x	x	x		x	x	

CIRAD Guadeloupe site de Duclos	France		x				x		
Labeo Frank Duncombe	France		x				x	x	
Robert Koch Institute	Germany		x						x
Istituto Zooprofilattico Sperimentale Del Lazio E Toscana	Italy	x	x	x	x			x	
Istituto Zooprofilattico Sperimentale delle Venezie, Padova	Italy		x					x	
Istituto Zooprofilattico Sperimentale della Lombardia e Dell'Emilia Romagna IZSLER	Italy		x				x	x	
CReSA (Centre de Recerca en Sanitat Animal)	Spain		x				x	x	
CISA-INIA (Centro de Investigación en Sanidad Animal)	Spain		x	x			x	x	x
**Mediterranean and Balkan NRLs**									
Veterinary Institute, University of Sarajevo	Bosnia and Herzegovina		x					x	
Biopharma	Morrocco	x	x	x			x	x	
**Kit manufacturers**									
LSI	France	x	x	x			x		
IDVET	France		x				x	x	
IDEXX	France		x					x	
