# Supplementary material for: Improved reliability of serological tools for the diagnosis of West Nile fever in horses within Europe
Source: PLoS Negl Trop Dis. 2017 Sep 15;11(9):e0005936. doi: 10.1371/journal.pntd.0005936 (PMC5617233; doi:10.1371/journal.pntd.0005936)
Supplement: S2 File — (PDF) [file pntd.0005936.s002.pdf]

S2 file: Results of the 2010 WNV ILPT per participant (IgG, IgM and VNT)

2010 IgG results (A-Q : NRLs results)

		Negative sample				WNV Duplicate		WNV Serial 2-fold dilutions					Low WNV	WNV	Low WNV Duplicate		WNV
	**IgG/competition ELISA**	**S1**	**S2**	**S3**	**S4**	**S5 (1/20)**	**S6 (1/20)**	**S7 (1/40)**	**S8 (1/80)**	**S9 (1/160)**	**S10 (1/320)**	**S11 (1/640)**	**S12 (1/80)**	**S13 (1/20)**	**S14 (1/40)**	**S15 (1/40)**	**S16 (1/10)**
A	*IDScreen WNV-215	N	N	N	N	P	P	D	N	N	N	N	N	P	N	N	P
A	*IDScreen WNV-145	N	N	N	N	P	P	P	P	D	N	N	D	P	P	P	P
B	*IDScreen WNV-145	N	N	N	N	P	P	P	D	N	N	N	D	P	P	P	P
C	*IDScreen WNV -145	N	N	N	N	P	P	P	P	N	N	N	D	P	P	P	P
D	*IDScreen WNV -145	N	N	N	N	P	P	P	P	D	N	N	D	P	P	P	P
E	*IDScreen WNV -145	N	N	N	N	P	P	P	D	N	N	N	N	P	D	D	D
E	In house indirect IgG ELISA, inactivated virions	N	N	N	N	N	N	N	N	N	N	N	N	P	N	N	N
F	*IDScreen WNV-145	N	N	N	N	P	P	P	P	N	N	N	D	P	P	P	P
G	*IDScreen WNV -145	N	N	N	N	P	P	P	D	N	N	N	N	P	P	P	N
H	*IDScreen WNV-145	N	N	N	N	P***	P	P	D	N	N	N	N	P	P	P	D
I	*IDScreen WNV-145	N	N	N	N	P	P	P	P	N	N	N	D	P	P	P	P
J	*IDScreen WNV-215	N	N	N	N	P	P	P	D	N	N	N	N	P	D	D	P
K	*ID Screen WNV -215	N	N	N	N	P	P	P	D	N	N	N	N	P	N	N	P
L	*ID Screen WNV -215	N	N	N	N	P	P	D	D	N	N	N	N	P	D	D	P
M	*ID Screen WNV -215	N	N	N	N	P	P	D	N	N	N	N	N	P	D	N	P
N	*IDScreen WNV-215	N	N	N	N	P	P	D	N	N	N	N	N	P	N	N	D
O	*ID Screen WNV -215	N	N	N	N	P	P	P	D	N	N	N	N	P	N	D	D
P	*ID Screen WNV -215	N	N	N	N	P	P	D	N	N	N	N	N	P	N	N	P
Q	*IDScreen WNV	N	N	N	N	P	P	P	D	N	N	N	N	P	N	D	N
Q	**Ingezim WNV compaq	N	N	N	N	D	D	D	N	N	N	N	N	D	N	N	N
R	In house	N	N	N	N	P	P	P	D	D	D	N	N	P	P	P	N
S	*IDScreen WNV-215	N	N	N	N	P	P	D	N	N	N	N	N	P	N	N	D
T	*IDScreen WNV-215	N	N	N	N	P	P	P	P	D	N	N	N	P	P	P	P
U	In house competition ELISA, inactivated virions	N	P	N	N	P	P	N	N	N	N	N	N	P	N	N	P
	**ILPT accepted results**	**N**	**N**	**N**	**N**	**P**	**P**	**P/D**	**P/D/N**	**P/D/N**	**N**	**N**	**P/D/N**	**P**	**P/D/N**	**P/D/N**	**P/D**

* ID screen WNV competition kit (IDVet)

** Ingezim WNV Compaq kit (Ingenasa)

N: negative; P: positive, D: doubtful
In grey : unsatisfactory results
*** Differing results on duplicate samples

2010 VNT results (NRLs results)

		Negative sample				WNV Duplicate		WNV Serial 2-fold dilutions					Low WNV	WNV	Low WNV Duplicate		WNV
	**Method**	**S1**	**S2**	**S3**	**S4**	**S5 (1/20)**	**S6 (1/20)**	**S7 (1/40)**	**S8 (1/80)**	**S9 (1/160)**	**S10 (1/320)**	**S11 (1/640)**	**S12 (1/80)**	**S13 1/20)**	**S14 (1/40)**	**S15 (1/40)**	**S16 (1/10)**
A	PRNT 90 (Vero cells, Is98 strain)	**N**	**N**	**N**	**N**	**N**	**N**	**N**	**N**	**N**	**N**	**N**	**N**	**N**	**P**	**P**	**N**
B	MNT (Vero cells)	**N**	**N**	**N**	**N**	**N**	**N**	**N**	**N**	**N**	**N**	**N**	**N**	**N**	**D**	**P**	**N**
E	PRNT 90+MNT (Vero cells, Eg 101 strain)	**N**	**N**	**N**	**N**	**N**	**N**	**N**	**N**	**N**	**N**	**N**	**N**	**N**	**D**	**D**	**N**
I	MNT (Vero cells)	**N**	**N**	**N**	**N**	**N**	**N**	**N**	**N**	**N**	**N**	**N**	**N**	**N**	**N**	**N**	**N**
K	MNT (Vero cells)	**N**	**N**	**N**	**N**	**N**	**N**	**N**	**N**	**N**	**N**	**N**	**N**	**N**	**P**	**P**	**N**
L	MNT (Vero cells)	**N**	**N**	**N**	**N**	**P**	**P**	**P**	**N**	**N**	**N**	**N**	**N**	**P**	**P**	**P**	**N**
P	MNT (Vero cells, NY99 or Ug strains)	**N**	**N**	**N**	**N**	**N**	**N**	**N**	**N**	**N**	**N**	**N**	**N**	**N**	**N**	**N**	**N**
Q	MNT (Vero cells)	**N**	**N**	**N**	**N**	**N**	**N**	**N**	**N**	**N**	**N**	**N**	**N**	**N**	**N**	**N**	**N**
	**ILPT accepted results**	**N**	**N**	**N**	**N**	**P/D/N**	**P/D/N**	**P/D/N**	**N**	**N**	**N**	**N**	**N**	**P/D/N**	**P/D**	**P/D**	**N**

N: negative; P: positive, D: doubtful
In grey : unsatisfactory results

2010 IgM results (A-K : NRLs results)

		Negative sample				WNV Duplicate		WNV Serial 2-fold dilutions					Low WNV	WNV	Low WNV Duplicate		WNV
	**Kits**	**S1**	**S2**	**S3**	**S4**	**S5 (1/20)**	**S6 (1/20)**	**S7 (1/40)**	**S8 (1/80)**	**S9 (1/160)**	**S10 (1/320)**	**S11 (1/640)**	**S12 (1/80)**	**S13 (1/20)**	**S14 (1/40)**	**S15 (1/40)**	**S16 (1/10)**
A	in house IgM ELISA	**N**	**N**	**N**	**N**	**N**	**N**	**N**	**N**	**N**	**N**	**N**	**N**	**N**	**P**	**P**	**N**
E	*IDEXX WNV IgM	**N**	**N**	**N**	**N**	**N**	**N**	**N**	**N**	**N**	**N**	**N**	**N**	**N**	**P**	**P**	**N**
F	*IDEXX WNV IgM - 149	**N**	**N**	**N**	**N**	**N**	**N**	**N**	**N**	**N**	**N**	**N**	**N**	**N**	**P**	**P**	**N**
K	*IDEXX WNV IgM - 0053	**N**	**N**	**N**	**N**	**N**	**N**	**N**	**N**	**N**	**N**	**N**	**N**	**N**	**P**	**P**	**N**
R	*IDEXX WNV IgM - 0053	**N**	**N**	**N**	**N**	**N**	**N**	**N**	**N**	**N**	**N**	**N**	**N**	**N**	**P**	**P**	**N**
T	in house IgM ELISA	**N**	**N**	**N**	**N**	**N**	**N**	**N**	**N**	**N**	**N**	**N**	**N**	**N**	**P**	**P**	**N**
	**ILPT accepted results**	**N**	**N**	**N**	**N**	**N**	**N**	**N**	**N**	**N**	**N**	**N**	**N**	**N**	**P**	**P**	**N**

*IDEXX IgM WNV Ab kit

N: negative; P: positive, D: doubtful
