# Supplementary material for: Improved reliability of serological tools for the diagnosis of West Nile fever in horses within Europe
Source: PLoS Negl Trop Dis. 2017 Sep 15;11(9):e0005936. doi: 10.1371/journal.pntd.0005936 (PMC5617233; doi:10.1371/journal.pntd.0005936)
Supplement: S3 File — (PDF) [file pntd.0005936.s003.pdf]

S3 file: Results of the 2013 WNV ILPT per participant (IgG, IgM and VNT)

2013 IgG results (A-Y: NRLs results)

		Negative	Kinetics of WNV lineage 1 antibody response			WNV Duplicate		Serial dilutions						Related flaviviruses		
**2013 code number**	**kits**	**S1**	**S2 8dpi**	**S3 21dpi**	**S4 35dpi**	**S5 (1/6)**	**S6 (1/6)**	**S7 (1/6)**	**S8 (1/24)**	**S9 (1/48)**	**S10 (1/384)**	**S11 (1/384)**	**S12 (1/536)**	**S13 USUV**	**S14 JEV**	**S15 TBEV**
A	*ID Screen WNV -460	N	N	P	P	P	P	P	P	P	N	N	N	P	P	P
B	*ID Screen WNV -334	N	N	P	P	P	P	P	P	P	N	N	N	P	P	P
C	*ID Screen WNV -334	N	N	P	P	P	P	P	P	P	N	N	N	P	P	P
D	*ID Screen WNV -334	N	N	P	P	P	P	P	P	P	N	N	N	P	P	P
E	*ID Screen WNV -460	N	N	P	P	P	P	P	P	P	N	N	N	P	P	P
E	In house WNV indirect ELISA, inactivated virions	N	N	P	P	P	P	P	D	P	N	N	N	N	N	N
F	*ID Screen WNV -334	N	N	P	P	P	P	P	P	P	N	N	N	P	P	P
G	*ID Screen WNV -334	N	N	P	P	P	P	P	P	P	N	N	N	P	P	P
H	*ID Screen WNV -145	N	D	P	P	P	P	P	P	P	N	N	N	P	P	P
I	** Ingezim West Nile -210213	N	P	P	P	P	P	P	P	P	N	N	N	P	P	N
I	*ID Screen WNV -334	N	N	P	P	P	P	P	P	P	N	N	N	P	P	P
J	*ID Screen WNV -460	N	N	P	P	P	P	P	P	P	N	N	N	P	P	P
K	*ID Screen WNV -460	N	N	P	P	P	P	P	P	P	N	N	N	P	P	P
L	**Ingezim West Nile -70812	N	P	P	P	P	P	P	P	P	N	N	N	D	P	N
M	*ID Screen WNV -334	N	N	P	P	P	P	P	P	P	N	N	N	P	P	P
N	*ID Screen WNV -460	N	N	P	P	P	P	P	P	P	N	N	N	P	P	P
O	*ID Screen WNV -334	N	N	P	P	P	P	P	P	P	N	N	N	P	P	P
P	*ID Screen WNV -334	N	N	P	P	P	P	P	P	P	N	N	N	P	P	P
V	*ID Screen WNV -334	N	N	P	P	P	P	P	P	P	N	N	N	P	P	P
W	*ID Screen WNV -334	N	N	P	P	P	P	P	D	P	N	N	N	P	P	P
X	*ID Screen WNV -334	N	N	P	P	P	P	P	P	P	N	N	N	P	P	P
Y	*ID Screen WNV -460	N	N	P	P	P	P	P	P	P	N	N	N	P	P	P
AA	*ID Screen WNV -460	N	N	P	P	P	P	P	P	P	N	N	N	D	P	D
AB	In house competition ELISA, inactivated cell supernatants	N	P	P	P	P	P	P	P	P	N	N	N	N	N	N
AC	*ID Screen WNV -460	N	N	P	P	P	P	P	P	P	N	N	N	P	P	P
AD	In house NS1 inhibition ELISA	N	N	P	P	P	P	P	P	P	N	N	N	N	P	N
AE	**Ingezim West Nile -70812	N	P	P	P	P	P	P	P	P	N	N	N	D	P	N
AF	*ID Screen WNV -334	N	N	P	P	P	P	P	P	P	N	N	N	P	P	P
AG	*ID Screen WNV -460	N	N	P	P	P	P	P	P	P	N	N	N	P	P	P
AH	*ID Screen WNV -460	N	N	P	P	P	P	P	P	P	N	N	N	P	P	P
AI	*ID Screen WNV -460	N	D	P	P	P	P	P	P	P	N	N	N	P	P	P
	**ILPT accepted results**	**N**	**P/D/N**	**P**	**P**	**P**	**P**	**P**	**P**	**P**	**N**	**N**	**N**	**P/D/N**	**P/D/N**	**P/D/N**

* ID screenWNV competition kit (IDVet)
** IngezimWNV Compaq kit (Ingenasa)

N: negative; P: positive, D: doubtful
In grey : unsatisfactory results

2013 IgM results (A-Z: NRLs results)

		Negative	Kinetics of WNV lineage 1 antibody response			WNV Duplicate		Serial dilutions						Related flaviviruses		
**2013 code number**	**Kits**	**S1**	**S2 8dpi**	**S3 21dpi**	**S4 35dpi**	**S5 (1/6)**	**S6 (1/6)**	**S7 (1/6)**	**S8 (1/24)**	**S9 (1/48)**	**S10 (1/384)**	**S11 (1/384)**	**S12 (1/536)**	**S13 USUV**	**S14 JEV**	**S15 TBEV**
A	*ID Screen West Nile IgM Capture -438	**N**	**P**	**P**	**P**	**N**	**N**	**P**	**N**	**N**	**N**	**N**	**N**	**N**	**N**	**N**
E	**IDEXX IgM WNV Ab test -2227	**N**	**P**	**P**	**N**	**N**	**N**	**P**	**D**	**D**	**N**	**N**	**N**	**N**	**N**	**N**
G	*ID Screen West Nile IgM -438	**N**	**P**	**P**	**N**	**N**	**N**	**P**	**N**	**N**	**N**	**N**	**N**	**N**	**N**	**N**
I	**IDEXX IgM WNV Ab test -3071	**N**	**P**	**P**	**D**	**N**	**N**	**P**	**D**	**D**	**N**	**N**	**N**	**N**	**N**	**N**
K	**IDEXX IgM WNV Ab test -2227	**N**	**P**	**P**	**D**	**N**	**N**	**P**	**N**	**D**	**N**	**N**	**N**	**N**	**N**	**N**
L	**IDEXX IgM WNV Ab test -2227	**N**	**P**	**P**	**D**	**N**	**N**	**P**	**D**	**N**	**N**	**N**	**N**	**N**	**N**	**N**
N	*ID Screen West Nile IgM -438	**N**	**P**	**P**	**N**	**N**	**N**	**P**	**N**	**N**	**N**	**N**	**N**	**N**	**N**	**N**
O	**IDEXX IgM WNV Ab test -2227	**N**	**P**	**P**	**D**	**N**	**N**	**P**	**D**	**N**	**N**	**N**	**N**	**N**	**N**	**N**
P	*ID Screen West Nile IgM -438	**N**	**P**	**P**	**D**	**N**	**N**	**P**	**N**	**N**	**N**	**N**	**N**	**N**	**N**	**N**
V	*ID Screen West Nile IgM -438	**N**	**P**	**P**	**P**	**N**	**N**	**P**	**N**	**N**	**N**	**N**	**N**	**N**	**N**	**N**
W	*ID Screen West Nile IgM -438	**N**	**P**	**P**	**N**	**N**	**N**	**P**	**N**	**N**	**N**	**N**	**N**	**N**	**N**	**N**
X	*ID Screen West Nile IgM -319	**N**	**P**	**P**	**N**	**N**	**N**	**P**	**N**	**N**	**N**	**N**	**N**	**N**	**N**	**N**
Z	*ID Screen West Nile IgM -668	**N**	**P**	**P**	**N**	**N**	**N**	**D**	**N**	**N**	**N**	**N**	**N**	**N**	**N**	**N**
AI	*ID Screen West Nile IgM -438	**N**	**P**	**P**	**N**	**N**	**N**	**P**	**N**	**N**	**N**	**N**	**N**	**N**	**N**	**N**
AA	*ID Screen West Nile IgM -438	**N**	**P**	**P**	**D**	**N**	**N**	**P**	**N**	**N**	**N**	**N**	**N**	**N**	**N**	**N**
AB	In-house IgM Capture ELISA	**N**	**P**	**P**	**D**	**N**	**N**	**P**	**N**	**N**	**N**	**N**	**N**	**N**	**N**	**N**
AC	*ID Screen West Nile IgM -438	**N**	**P**	**P**	**N**	**N**	**N**	**D**	**N**	**N**	**N**	**N**	**N**	**N**	**N**	**N**
AE	***Ingezim West Nile IgM -0612	**N**	**P**	**P**	**P**	**P**	**P**	**P**	**P**	**P**	**N**	**N**	**N**	**N**	**N**	**N**
AF	*ID Screen West Nile IgM -319	**N**	**P**	**P**	**D**	**N**	**N**	**P**	**N**	**N**	**N**	**N**	**N**	**N**	**N**	**N**
AG	*ID Screen West Nile IgM -438	**N**	**P**	**P**	**D**	**N**	**N**	**P**	**N**	**N**	**N**	**N**	**N**	**N**	**N**	**N**
AJ	*ID Screen West Nile IgM -438	**N**	**P**	**P**	**N**	**N**	**N**	**P**	**N**	**N**	**N**	**N**	**N**	**N**	**N**	**N**
AK	**IDEXX IgM WNV Ab test -2227	**N**	**P**	**P**	**P**	**N**	**N**	**P**	**D**	**D**	**N**	**N**	**N**	**N**	**N**	**N**
AL	*ID Screen West Nile IgM -438	**N**	**P**	**P**	**N**	**N**	**N**	**N**	**N**	**N**	**N**	**N**	**N**	**N**	**N**	**N**
AL	**IDEXX IgM WNV Ab test -3071	**N**	**P**	**P**	**P**	**N**	**N**	**P**	**N**	**D**	**N**	**N**	**N**	**N**	**N**	**N**
AM	*ID Screen West Nile IgM -668	**N**	**P**	**P**	**D**	**N**	**N**	**D**	**N**	**N**	**N**	**N**	**N**	**N**	**N**	**N**
	**ILPT accepted results**	**N**	**P**	**P**	**P/D/N**	**P/D/N**	**P/D/N**	**P/D/N**	**P/D/N**	**P/D/N**	**N**	**N**	**N**	**N**	**N**	**N**

* ID screen WNV IgM capture kit

**IDEXX IgM WNV Ab kit

***Ingezim WNV IgM capture Kit

N: negative; P: positive, D: doubtful

2013 VNT results (A-P: NRLs results)

		Negative	Kinetics of WNV lineage 1 antibody response			WNV Duplicate		Serial dilutions						Related flaviviruses		
**2013 code number**		**S1**	**S2 8dpi**	**S3 21dpi**	**S4 35dpi**	**S5 (1/6)**	**S6 (1/6)**	**S7 (1/6)**	**S8 (1/24)**	**S9 (1/48)**	**S10 (1/384)**	**S11 (1/384)**	**S12 (1/536)**	**S13 USUV**	**S14 JEV**	**S15 TBEV**
**A**	WNV MNT (Vero, Is98)	**N**	**P**	**P**	**P**	**P**	**P**	**P**	**P**	**P**	**N**	**N**	**N**	**N**	**N**	**N**
	USUV MNT (Vero, SAAR 1776)	**N**	**N**	**P**	**P**	**N**	**N**	**N**	**N**	**N**	**N**	**N**	**N**	**P**	**P**	**N**
	JEV MNT (Vero, Nakayama)	**N**	**N**	**P**	**P**	**N**	**N**	**P**	**N**	**N**	**N**	**N**	**N**	**N**	**N**	**P**
	TBEV MNT (Vero, Hypr)	**N**	**N**	**N**	**N**	**N**	**N**	**N**	**N**	**N**	**N**	**N**	**N**	**N**	**P**	**N**
**B**	WNV MNT (Vero, Is98)	**N**	**N**	**P**	**P**	**P**	**P**	**P**	**P**	**P**	**N**	**N**	**N**	**N**	**N**	**N**
**E**	WNV MNT (Vero, Eg101)	**N**	**N**	**P**	**P**	**P**	**P**	**P**	**P**	**P**	**N**	**N**	**N**	**N**	**N**	**N**
**I**	WNV MNT (Vero, Eg101)	**N**	**N**	**N**	**P**	**D**	**D**	N	**N**	**N**	**N**	**N**	**N**	**N**	**N**	**N**
**K**	WNV MNT (Vero, NY99)	**N**	**P**	**P**	**P**	**P**	**P**	**P**	**P**	**P**	**N**	**N**	**N**	**N**	**N**	**N**
**L**	WNV MNT (Vero, Eg101)	**N**	**N**	**P**	**P**	**P**	**P**	**P**	**P**	**P**	**N**	**N**	**N**	**N**	**N**	**N**
**P**	WNV MNT (Vero, NY99)	**N**	**N**	**N**	**P**	**P**	**P**	N	**N**	**N**	**N**	**N**	**N**	**N**	**N**	**N**
**AN**	WNV MNT (Vero, IS98)	**N**	**N**	**P**	**P**	**P**	**P**	N	**N**	**N**	N	N	**N**	**N**	**N**	**N**
**AO**	WNV MNT (Vero, Eg101)	**N**	**P**	**P**	**P**	**P**	**P**	**P**	**N**	**P**	**N**	**N**	**N**	**N**	**N**	**N**
	**ILPT accepted results (WNV)**	**N**	**P/D/N**	**P**	**P**	**P/D**	**P/D**	**P**	**P/D/N**	**P/D/N**	**N**	**N**	**N**	**N**	**N**	**N**

N: negative; P: positive, D: doubtful

In grey: unsatisfactory results
