# Supplementary material for: Improved reliability of serological tools for the diagnosis of West Nile fever in horses within Europe
Source: PLoS Negl Trop Dis. 2017 Sep 15;11(9):e0005936. doi: 10.1371/journal.pntd.0005936 (PMC5617233; doi:10.1371/journal.pntd.0005936)
Supplement: S1 Fig — %S/P was calculated according to the manufacturer’s instructions. The threshold value for considering a serum as positive was % S/P greater than or equal to 45%. (PDF) [file pntd.0005936.s004.pdf]

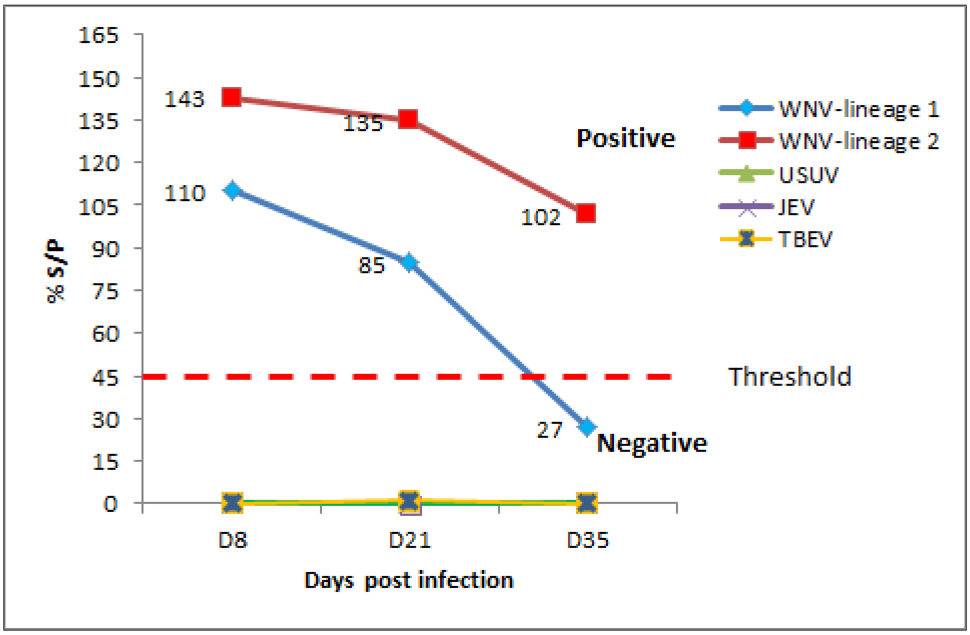

S1 Fig: Reference equine sera sampled from ponies infected by different flaviviruses (WNV lineage 1, WNV lineage 2, USUV, JEV and TBEV) collected on different days (D) after infection (D8, D21 and D35) and tested by ID screen WNV IgM capture kit. %S/P was calculated according to the manufacturer’s instructions. The threshold value for considering a serum as positive was % S/P greater than or equal to 45%.
